# RELATIONSHIP DISSOLUTION AND BIOLOGICAL AGING IN MID LIFE

**DOI:** 10.1093/geroni/igad104.0309

**Published:** 2023-12-21

**Authors:** Kyle Bourassa, Grace Brennan, Avshalom Caspi, Terrie Moffitt

**Affiliations:** Durham VA Medical Center, Durham, North Carolina, United States; Duke University, Durham, North Carolina, United States; Duke University, Durham, North Carolina, United States; Duke University, Durham, North Carolina, United States

## Abstract

Several decades of empirical research has shown that people who experience relationship dissolution—such as romantic breakups, divorce, and bereavement—have increased risk for poor health across a range of disease outcomes. However, it remains less clear which physiological pathways might best explain links between relationship dissolution and later health. One candidate mechanism that might help explain this wide range of observed health outcomes is accelerated biological aging, which is theorized to represent a common cause of age-related chronic diseases, disability, and early mortality. The current study examined whether relationship dissolution, measured across a 20-year period of adulthood, was associated with biological age in midlife using participants (n = 910) from the Dunedin Study, a birth cohort assessed over four decades of life. Relationship dissolution was assessed using a count of romantic breakups from age 26 until age 44 from life history calendars. Biological aging was assessed using a midlife aging factor derived from four validated measures of aging—the Pace of Aging, gait speed, BrainAGE, and facial age—assessed at age 45. We found that people with more breakups in adulthood had more advanced biological age in midlife, β = 0.10, 95% CI [0.04, 0.16], p = .003. This association remained when controlling for adversity and poor health in childhood, as well as disadvantaged socioeconomic origins and time spent in romantic relationships, β = 0.09, 95% CI [0.03, 0.15], p = .007. The results suggest accelerated aging may be one physiological mechanism linking relationship dissolution to poor health.

